# Characterization of celiac disease related oat proteins: bases for the development of high quality oat varieties suitable for celiac patients

**DOI:** 10.1038/srep42588

**Published:** 2017-02-17

**Authors:** María J. Giménez, Ana Real, M. Dolores García-Molina, Carolina Sousa, Francisco Barro

**Affiliations:** 1 Instituto de Agricultura Sostenible, CSIC, Córdoba, Spain; 2 INSERM U1218 “ACTION”, Bordeaux University, Bordeaux Cedex, France; 3 Departamento de Microbiología y Parasitología, Facultad de Farmacia, Universidad de Sevilla, Sevilla, Spain

## Abstract

Some studies have suggested that the immunogenicity of oats depends on the cultivar. RP-HPLC has been proposed as a useful technique to select varieties of oats with reduced immunogenicity. The aim of this study was to identify both the avenin protein patterns associated with low gluten content and the available variability for the development of new non-toxic oat cultivars. The peaks of alcohol-soluble avenins of a collection of landraces and cultivars of oats have been characterized based on the RP-HPLC elution times. The immunotoxicity of oat varieties for patients with celiac disease (CD) has been tested using a competitive ELISA based on G12 monoclonal antibody. The oat lines show, on average, seven avenin peaks giving profiles with certain similarities. Based on this similarity, most of the accessions have been grouped into avenin patterns. The variability of RP-HPLC profiles of the collection is great, but not sufficient to uniquely identify the different varieties of the set. Overall, the immunogenicity of the collection is less than 20 ppm. However, there is a different distribution of toxicity ranges between the different peak patterns. We conclude that the RP-HPLC technique is useful to establish groups of varieties differing in degree of toxicity for CD patients.

Cultivated oats are hexaploid cereals belonging to the genus *Avena sativa* L., which is found worldwide in almost all agricultural environments. Oats have a relatively minor status among cereals because they are more difficult to process and, due to their high lipid content and lipase activity, oat products have a greater instability. Compared with the major food grain cereals consumed globally, oats have a similar nutrition profile, with the exception of a higher amount of protein, some nutrients, and most notably a high fiber content in the form of betaglucan. Because oats are more likely to be consumed as whole grain, recommendations that emphasize increasing oat consumption specifically, while increasing overall whole grain intake, would be expected to have a positive effect on human health^1^. In that sense, reports of the possible blood cholesterol lowering effect of oat bran have increased the popularity of its use for human food in developed countries^2^. Another reason for the increasing interest in oats concerns the quality and quantity of avenins, the second major class of seed oat proteins. Avenins are prolamins, a family of closely related proline- and glutamine-rich proteins^3^, amino acids involved in the triggering of celiac disease (CD), an autoimmune disorder of the gastrointestinal tract. Oats are considered non-immunogenic or less immunogenic than other cereals^4^,^5^,^6^, but there are studies demonstrating that oat intolerance does exist in some patients with CD^7^,^8^,^9^,^10^. The introduction of oats in the gluten-free diet has been a topic of debate in recent years^11^,^12^, as some studies suggest that the oat immunogenicity depends on the cultivar used^13^,^14^. In southern Europe, at the beginning of the decade, oats represented only about six percent of the cereal-cultivated area and less than 3% of cereal production, being consumed almost exclusively as livestock feed^15^. The yields in this area were also much lower than those obtained in northern Europe. Due to the potential benefits of the inclusion of oats in the diets of both celiac and general populations, a program of breeding for oats was started in Andalusia (Spain) in order to obtain more-productive gluten-free oat varieties. Differences have been found in the immunogenic profile between oat varieties, suggesting that the selection and breeding of oat genotypes that have no risk for CD patients may be feasible^16^. The identification of lines with low gluten content, and therefore low immunotoxicity for CD patients, adapted to many environmental conditions is a first step in a breeding program for gluten-free oats^17^, and, for that purpose, having tools to determine the availability of genetic variability is essential In a breeding program, molecular and/or genetic markers associated to the characters to be incorporated in a variety facilitate the selection process and, therefore, enable a more efficient improvement. Oat avenins could play a role similar to that of the endosperm storage proteins of wheat, which have traditionally been used with a dual purpose since, on the one hand, the electrophoretic profiles of gliadin are useful for varietal identification, secondly, as markers associated to quality parameters. A combined method using electrophoresis and reversed-phase high performance liquid chromatography (RP-HPLC) of oat avenins has been reported^18^ and the usefulness of the RP-HPLC for varietal identification of oats has been clearly demonstrated^19^. Moreover, it has been suggested that this technique would be useful for selecting oat varieties with reduced immunogenicity for CD patients^20^. The aim of this study was to determine the variability of important agronomic traits and avenin patterns in a collection of accessions from different origins, and establish the relationship between profiles and gluten toxicity for CD patients. The study will facilitate the selection of outstanding parents adapted to Mediterranean conditions to develop oat varieties suitable for a gluten-free diet.

## Results

### Characterization of the oat accessions by their origin

**Figure 1:**
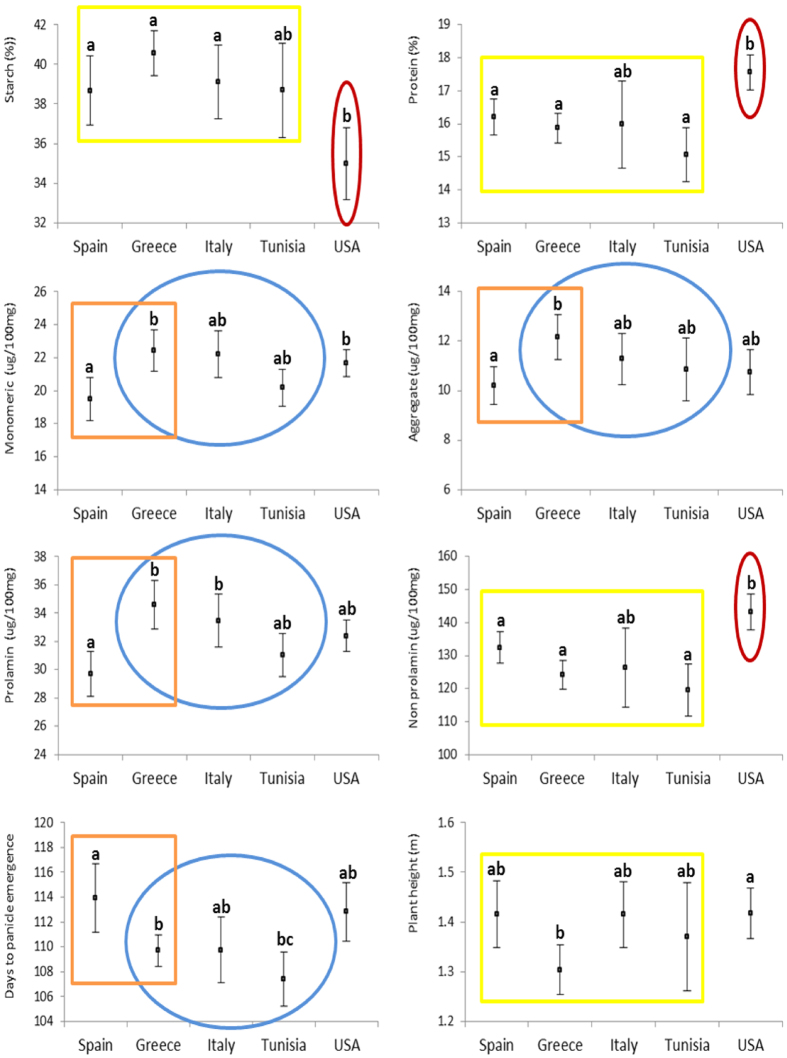
Means and 95% CIs for agronomic plant traits and flour quality composition of oat accessions grouped by their origin. Different letters mean a significant difference (p < 0.05). Yellow rectangles indicate no significant differences among Mediterranean accessions; Blue ovals and orange squares highlight differences between Spanish and Greek accessions. Red ovals indicate that a mean is significantly different from at least other three group means.

The accessions included in this study come from five countries: four of them from the Mediterranean area (Spain, Greece, Italy, and Tunisia) and the fifth, the USA. The overall mean of the evaluated parameters of all these lines as well as the means of the subsets according to their origin are shown in Table 1 (raw data can be found in Supplementary Table S1). When the mean values of each group were compared with that of the whole set, the group of lines from Greece presented more differences than any other, since that group was significantly earlier and with lower plant height, and the composition of the flour also significantly differed for all parameters except protein content. The group from Tunisia was also earlier, while the USA group was the second most deviating from the average, with lower and higher levels of starch and protein, respectively, in flour. Comparison of the means between the groups of accessions from Greece, Italy, and Tunisia showed overlapping confidence intervals (95% CI) for all variables, both plant characteristics and the flour composition (Fig. 1). The oat groups from Spain and Greece significantly differed for the flowering time and prolamin contents, with the amounts of monomeric and aggregate prolamin higher in the second group. The starch, total protein and non-prolamin contents, as well as the protein to starch ratio, of the subset of accessions from the USA were significantly different from those of at least three of the groups from the Mediterranean area (Supplementary Fig. S1).

### RP-HPLC peak profiles of monomeric avenins

**Figure 2:**
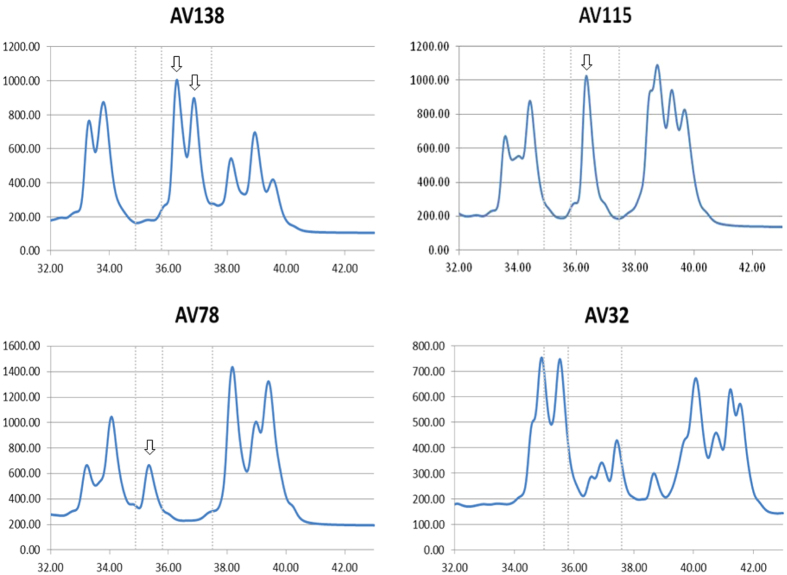
Examples of chromatograms of the RP-HPLC oat groups. AV138 (Pattern I); AV115 (Pattern II); AV78 (Pattern III); AV32 (Other). Arrows indicate the peaks used as reference for establishing the groups within each range (dotted line) of elution time.

Aliquots of prolamin extracts of oat lines were subjected to RP-HPLC. The monomeric components consisted of two or three major reproducible groups of peaks that were eluted close together in the hydrophobic range (33–43 min). The number of peaks varied among accessions (seven peaks on average), with AV132 and AV118 having the highest and the lowest number of peaks, respectively. In HPLC, the area under the peak indicates the amount of the compound present in the sample. In our set some lines sharing the same avenin profile, but with different relative amount of each of them were found (Supplementary Fig. S2). Although it could be interpreted that these actually correspond to two different proteins that elute at the same time, we have considered them as the same for ease of analysis. There were accessions with very similar RP-HPLC profiles. However, a great variability was found in the collection, and only a few of them shared identical peaks. After a visual inspection of the chromatograms, the accessions were separated into four groups (patterns) based on the following: no peaks between 35.4 min and 35.9 min and with two peaks in the range 35.9–37.5 min (Pattern I) or one peak in the range 35.9–36.7 min (Pattern II); presence of peaks in the range 35.4–35.9 min and no major peaks in the range 35.9–37.5 min (Pattern III). The fourth group (Other) comprised those accessions whose chromatograms could not be considered as belonging to any of the above groups. Examples of the patterns are presented in Fig. 2. According to the origin of the lines, the frequency of the patterns differed (Table 2). Thus, pattern I is more common in the eastern Mediterranean, while pattern III is barely represented in the whole Mediterranean basin. For the USA, all accessions can be classified as belonging to pattern I, II or III, patterns II and III being the most abundant. The commercial cultivars present all profiles though pattern II is the most frequent. The group of cultivars from Spain presented the highest variation, since most of them were included in group IV, the group most variable in both number and types of peaks according to the time of elution.

### Characterization of the oat accessions by their RP-HPLC pattern

The plant and flour characteristics of accessions of the whole set, grouped by patterns, were analyzed. The overall mean of the evaluated parameters of all these lines, as well as the means of subsets according to their peak profiles are shown in Table 3. The group of lines of pattern I presented differences from the set for all the variables, since that group was significantly earlier and with lower plant height, and had a different flour composition. Nevertheless, relating the flour component ratios, the prolamin to protein ratio was the only one different from the average. Lines with pattern III presented later panicle emergence and greater plant height. In addition, starch and protein contents were significantly lower and higher, respectively, and the prolamin to protein ratio was lower. Pattern II mean values differed significantly from the overall mean only for the ratios protein to starch, monomeric to aggregate prolamin content, and monomeric to total prolamin content. The between-group comparisons showed that the accessions sharing pattern I were earlier in time of flowering and had a higher starch content than the groups with pattern II and III, whereas the plant height of the lines with pattern III was greater than in those with patterns I and II (Supplementary Fig. S3). The prolamin to protein ratio of the pattern I group was significantly different from those of the other groups, and starch to protein of the pattern I group did not overlap with that of the pattern III group (Supplementary Fig. S4).

### Gluten content

**Figure 3:**
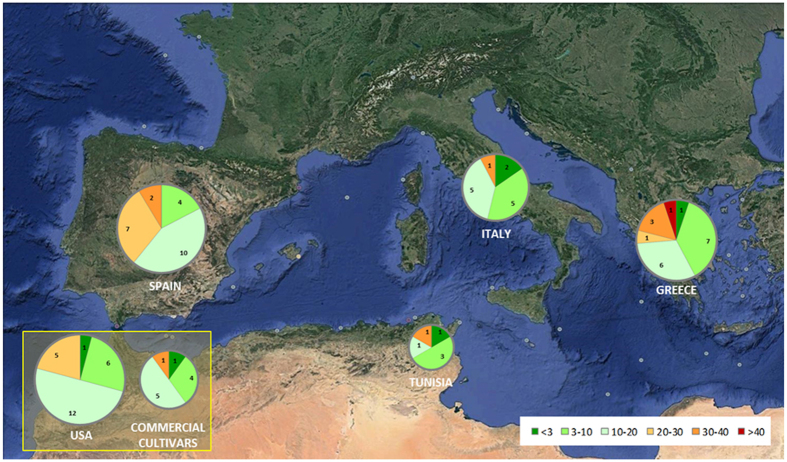
Geographical distribution of the set of landraces and commercial cultivars included in this study, grouped by range of gluten content (<3 ppm, 3–10 ppm, 10–20 ppm, 20–30 ppm, 30–40 ppm and >40 ppm). The area of the circles is proportional to the number of accessions. Numbers of accessions in black within each pie chart. Charts have been superimposed on a map from Google Earth (©2016 Google. Image: Landsat. Data: SIO, NOAA, US Navy, NGA, GEBCO).

**Figure 4:**
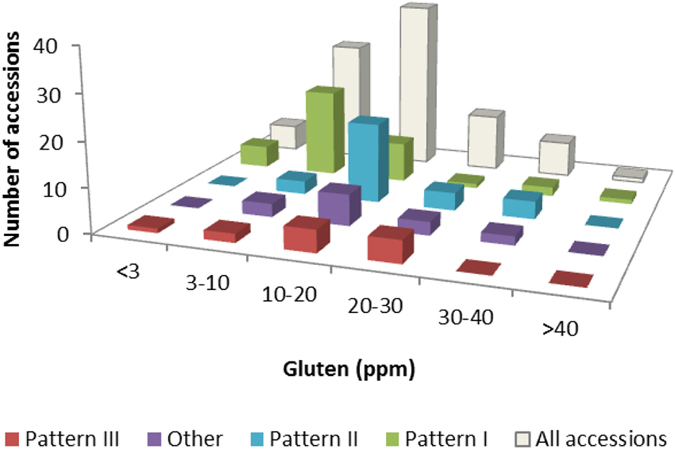
Graph of the distribution of the frequencies of ranges of gluten content for each RP-HPLC pattern.

The gluten content was determined by G12 antibody (moAb),which has a range of epitope recognition that includes prolamin peptides, involved in both the adaptive and innate immunological response in CD^20^,^21^,^22^,^23^. T-cell reactivity analysis and enzymatic detoxification by glutenase of the gluten show that the signal of this antibody is correlated with the potential toxicity of the sample for celiac patients^21^,^24^. The gluten content determined by competitive G12 ELISA was below 20 parts per million (ppm) in most of the oat accessions analyzed (73%) (raw data can be found in Supplementary Table S1), which can be regarded as “gluten-free” according to the Codex Alimentarius normative^25^. Furthermore, taking into account this limit, the potential immunotoxicity of the rest of the lines studied could be considered low as only one showed a gluten content two times above the limit. With respect to geographical origin, the frequency of non-toxic oats is highest in Tunisia (5 out of 6 accessions) and Italy (9 out of 10), while in Spain only 14 of 23 entries can be considered “gluten-free”. In addition, accessions with a content less than or equal to 10 ppm most often originate in the eastern Mediterranean (Fig. 3). We have tried to establish a relationship between a qualitative aspect (chromatographic profile) and gluten content. The frequency distribution of gluten content ranges in pattern I is different from that in the other patterns (Fig. 4). When comparing the frequency distribution of the six classes of gluten content (<3, 3–10, 10–20, 20–30, 30–40 and >40 ppm) for each group with the whole set of varieties, significant differences were found only for pattern I (Table 4A). Five of the six lines with gluten content below 3 ppm belong to this group, and the accessions with gluten content below 10 ppm are also more frequent than in the other groups. However, the AV82 entry of Greek origin and pattern I, is the only one whose gluten content is higher than 40 ppm. Considering only two classes of gluten content, below or above 10 ppm, the patterns I and II were significantly different from the entire collection, their accessions presenting a higher and a lower frequency of gluten content below 10 ppm than expected, respectively (Table 4B).

## Discussion

The immunogenicity of oat varieties evaluated by competitive G12 ELISA was low (only one input had more than 40 ppm of gluten) and most of them could be considered “gluten-free” (in the range 3–20 ppm) according to the European normative^25^. As mentioned above some studies have suggested that there is great variability for immunotoxicity in oats^13^,^14^. In this paper, we have analyzed a much larger number of lines than in previous works so, notwithstanding that there are some toxic cultivars, we can conclude that, in general, toxicity of the species is low. The introduction of oats in the gluten-free diet has been recommended in numerous studies (reviewed in ref. 26) for both children and adults^27^,^28^,^29^,^30^,^31^. The apparent contradiction between those works and the results of other studies^8^,^10^ could be due to the use of oat varieties included in the small percentage (less than 1% in our collection) that exceed 40 ppm. An amount of about 80 grams of rolled oats is the usually recommended daily dose for healthy eating. Even in the case of an intake of grain from oat lines with a gluten content of 20–40 ppm, the total amount of gluten would be as much as 3.2 mg, well below the amount of 50 mg/d established by^32^ as a safety margin for the variable gluten sensitivity and dietary habits of patients and lower, in any case, than the limit of ¡ 10 mg/d proposed by other authors^33^. We were able to find an association between the presence and/or absence of peaks in the middle of the chromatogram and gluten content. However, we cannot conclude that these proteins are the only determinants of immunogenicity as some of the accessions to the pattern associated with lower toxicity have high levels of gluten. The number of monomeric avenins separated by RP-HPLC coincides with that found by other authors^3^,^34^ so we can assume that each peak corresponds to a different protein. One explanation for the differences in the toxicity could be the variation in the amount of protein. The height of the peaks is not always the same for the accessions belonging to identical chromatographic profile indicating differences in prolamin content, in general, in the sample. When what differs is the peak height of a specific avenin relative to the others present in the same chromatogram within a specific group, it could be due to a pattern of expression of the same gene depending on the genetic background, or the coincidence in time of elution of two different avenins. As it has been previously reported, the variation of potential immunotoxicity of oat cultivars could be due to differences in the degree of immunogenicity in their sequences^20^. Consequently, the coincidence of two different avenins should be confirmed by sequencing since the resolution of other techniques for protein separation such as electrophoresis is even lower^19^. Storage proteins of cereals have been widely used for variety identification and for the study of genetic diversity, and have received special attention, particularly wheat gliadin, because of their implications for celiac disease. A great variability for these proteins was reported previously but, although the oat lines studied in this paper showed differences in their chromatograms allowing the differentiation of many of the varieties, our data agree with those leading to the conclusion that avenin composition as a single trait is insufficient to differentiate between all oat varieties in a large germ plasm collection^34^. Nevertheless, enough genetic variation exists in this collection of landraces offering opportunities for breeding new cultivars, especially in the group of Spanish origin. Almost all accessions that have a toxicity below 3 ppm belong to the pattern I group, 3–10 ppm being the most frequent value. Therefore, although the RP-HPLC technique is not sufficient to uniquely identify the different varieties in the analyzed collection, it is useful for grouping the entries in patterns that differ in degree of toxicity, and can be a valuable tool for oat genetic breeding. Agronomic traits and adaptation to biotic and abiotic stress have been the targets of the local selection of crops. For wheat, little genetic association has been found between yield, its components, and prolamin genes as revealed by meta-QTL analysis^35^. However selection for resistance to diseases and pests, one of the major constraints of yield in crop plants, could have implied the selection of particular alleles of prolamins as genes for disease resistance are distributed in gene rich regions all over the wheat genome, including those in group 1 and group 6 where gliadin loci are encoded^36^,^37^. The close relationship between geographic distribution and some alleles of blocks of gliadins has been attributed to this fact^38^. We have found that patterns of avenins are not evenly distributed among countries of origin supporting previous works^39^. Linkage between oat prolamins and disease resistance genes has been reported^40^,^41^ and, thus, their co-variation under environmental and breeding pressure seems to have resulted in the non-random distribution of the avenin profiles. In conclusion, the evaluation of these patterns in collections of landraces of different origins, such as the one in this study, is therefore, valuable for searching genotypes both with adequate agronomic performance and less toxic ones.

## Methods

### Plant material

A collection of 132 oat accessions (*Avena sativa* L., 2n = 6x = 42), 120 landraces or old varieties from the collection of the ‘National Small Grains Collection’ of the United States Department of Agriculture (USDA, USA), and 12 commercial cultivars of oats commonly grown in Spain, were sown in Córdoba (Spain) in December 2012 (Supplementary Table S2). Plants were grown until full maturity in single rows 1 m long. Samples of hulled grain of each line were ground in a cyclone mill to obtain flour of a particle size of 100 microns.

### Characterization of agronomic and quality parameters

In order to assess the aptitude of the accessions for their cultivation in the south of Spain, the variables days to panicle emergence and plant height (cm) at maturity were recorded. Quality parameters of the flour such as moisture, starch and protein contents were determined following the official methods by the Laboratorio Agroalimentario of Córdoba.

### Reversed-phase high-performance liquid chromatography (RP-HPLC)

Alcohol soluble avenins (monomeric) and aggregate avenins (polymeric) were sequentially extracted as described for wheat gliadins and glutenins^42^ from 100 mg of oat flour. Briefly, the monomeric fraction was extracted stepwise three times with 670 microliters of 60% (v/v) ethanol, vortexing for 2 min at room temperature (RT), followed by incubation at RT for 10 min with shaking. The insoluble material from the previous step was used to obtain the polymeric fraction, which was extracted stepwise twice with 500 microliters of 50% (v/v) 1-propanol, 2 M urea, 0.05 M Tris-HCl (pH 7.5) and 2% (w/v) DTT, vortexing for 2 min at RT, and incubating for 15 min at 60 °C with shaking. Samples were centrifuged at 6,000 × g for 15 min, and the supernatants were collected and mixed together. Finally, samples were filtered through a 0.45 microns nylon filter (Teknokroma, Barcelona, Spain). Avenin extracts (80 microL) were applied to a 300SB-C8 reverse phase analytical column (4.6 × 250 mm, 5 microns particle size, 300 Amstrongs pore size, Agilent Technologies) using a 1200 Series Quaternary LC System liquid chromatograph (Agilent Technologies) with a DAD UV-V detector, as described in refs 43,44. Absorbance was monitored with the DAD UV-V module at 210 nm. The amounts of both fractions per 100 mg flour were determined using bovine serum albumin (BSA; BSA 98%, fraction V. Sigma-Aldrich, St Louis,MO, cat. no. A3294) as protein standard. All protein measures were corrected by the moisture content for each sample. Prolamin contents were calculated as the sum of monomeric and aggregate contents whereas non-prolamin contents were estimated as the difference between total protein contents and prolamin contents. RP-HPLC profiles of peaks of the alcohol soluble fraction were described for each accession, each peak being defined based on the elution time. The integration and the peaks detection procedures were automatically handled by the software. To determine the range of variation of the elution time of peaks, a control sample was repeatedly injected every ten samples.

### Determination of immunotoxicity

CD toxicity of 95 oat accessions was determined using a competitive ELISA based on the monoclonal antibody G12^21^. Gluten content values of whole oats were recorded as <3 ppm, 3–10 ppm, 10–20 ppm, 20–30 ppm, 30–40 ppm and >40 ppm.

### Data analysis

Arithmetic mean values and their 95% confidence interval were calculated for the variables days to panicle emergence and plant height, and for contents in starch, protein and protein fractions of the accessions grouped by their origin. Geometric mean values and their 95% confidence interval were also calculated for each origin for the ratios starch/protein, monomeric/aggregate and prolamin/protein. For each variable, the means of each group were compared with the overall mean of the whole set of lines. Differences between groups were considered significant when both means had non-overlapping 95% confidence intervals. After a visual inspection of the chromatograms the accessions were separated into groups based on the presence and/or absence of peaks eluted in the central region of the chromatogram. For the subsets of lines grouped by RP-HPLC, the mean values of the variables and their 95% confidence interval were calculated. The statistical significances were determined in the same way as for the groups by origin. Frequencies of toxic gluten content classes were calculated for each pattern, and a chi-square (*χ*^2^) test of goodness-of-fit was used to test whether or not the distribution of the classes for each group fitted the distribution of the whole set of accessions.

### Data availability

Accession codes and raw can be found in the archive SupplementaryInformation-OatsCeliac-Gimetal2016.pdf.

## Additional Information

**How to cite this article**: Giménez, M. J. *et al*. Characterization of celiac disease related oat proteins: bases for the development of high quality oat varieties suitable for celiac patients. *Sci. Rep.* **7**, 42588; doi: 10.1038/srep42588 (2017).

**Publisher's note:** Springer Nature remains neutral with regard to jurisdictional claims in published maps and institutional affiliations.

## Supplementary Material

Supplementary Information

## Figures and Tables

**Table 1:** Comparison of agronomic plant traits and flour quality component mean values of each group of origin to the overall mean (NS = no significant; *p < 0.05, **p < 0.01; ***p < 0.001).

Parameter	Overall	Spain	Greece	Italy	Tunisia	USA
n	120	29	28	19	10	34
Panicle emergence	111.4	113.9 NS	109.7**	109.7 NS	107.4**	112.8 NS
Plant height	1.38	1.42 NS	1.30**	1.41 NS	1.37 NS	1.42 NS
Starch	37.28	38.67 NS	40.58***	39.12 NS	38.69 NS	34.98*
Protein	16.07	16.21 NS	15.87 NS	15.98 NS	15.05*	17.56***
Monomeric	20.66	19.48 NS	22.45**	22.21*	20.17 NS	21.66*
Aggregate	10.65	10.21 NS	12.15**	11.27 NS	10.85 NS	10.73 NS
Prolamin	31.31	29.69*	34.60***	33.48*	31.03 NS	32.39 NS
Non prolamin	129.4	132.4 NS	124.1*	126.3 NS	119.5*	143.2***
Protein/Starch	0.43	0.42 NS	0.39 NS	0.40 NS	0.39*	0.51*
Monomeric/Aggregate	1.95	1.91 NS	1.86 NS	1.99 NS	1.88 NS	2.07 NS
Prolamin/Protein	0.20	0.18*	0.22*	0.21 NS	0.21 NS	0.18 NS

**Table 2:** Number of lines and Frequency of the patterns of RP-HPLC peaks of avenins according to their origin.

	Spain	Greece	Italy	Tunisia	USA	Cultivars	Total
Pattern I	6 (21%)	21 (75%)	12 (63%)	7 (70%)	7 (21%)	2 (17%)	55
Pattern II	5 (17%)	6 (22%)	4 (21%)	3 (30%)	14 (41%)	5 (42%)	37
Pattern III	4 (14%)	0 (0%)	2 (11%)	0 (0%)	13 (38%)	3 (25%)	22
Other	14 (48%)	1 (6%)	1 (5%)	0 (0%)	0 (0%)	2 (17%)	18
	29	28	19	10	34	12	132

**Table 3:** Comparison of the plant features and flour components mean values of each group of pattern (PI, PII, PIII and Other) to the overall mean (NS = no significant; *p < 0.05, **p < 0.01; ***p < 0.001).

	Overall	PI	PII	PIII	Other
n	122	54	33	19	16
Panicle emergence	112.7	108.5***	112.5 NS	115.5*	114.3 NS
Plant height	1.41	1.33***	1.36 NS	1.53***	1.43 NS
n	132	55	37	22	18
Starch	36.77	39.78***	35.07 NS	34.00*	38.25 NS
Protein	16.25	15.46**	16.28 NS	17.41*	15.84 NS
Monomeric	20.23	21.35**	20.94 NS	20.50 NS	18.13*
Aggregate	10.47	11.39**	9.90 NS	10.53 NS	10.08 NS
Prolamin	30.71	32.75**	30.84 NS	31.03 NS	28.21 NS
Non prolamin	131.7	121.8***	132.0 NS	143.1*	130.2 NS
Protein/Starch	0.43	0.39 NS	0.47*	0.52***	0.42 NS
Monomeric/Aggregate	1.97	1.89 NS	2.17**	1.96 NS	1.84 NS
Prolamin/Protein	0.19	0.21***	0.19 NS	0.18**	0.18*

**Table 4:** Chi-squared test for goodness of fit of the distribution of ranges of gluten content of (A) each RP-HPLC group to the one of the whole set of accessions, and (B) of accessions with less and more of 10 ppm of gluten content to the one of whole set of accession.

	Pattern I	Pattern II	Pattern III	Other	All
	O (E)	O (E)	O (E)	O (E)	O
(A)					
<3 ppm	5 (2.4)	0 (1.8)	1 (0.8)	0 (0.9)	6
3–10 ppm	20 (11.2)	3 (8.5)	2 (3.8)	3 (4.4)	28
10–20 ppm	9 (15.6)	18 (11.9)	5 (5.3)	7 (6.2)	39
20–30 ppm	1 (5.2)	4 (4.0)	5 (1.8)	3 (2.1)	13
30–40 ppm	2 (3.2)	4 (2.4)	0 (1.1)	2 (1.3)	8
>40 ppm	1 (0.4)	0 (0.3)	0 (0.1)	0 (0.2)	1
*χ*^2^	13.2	7.22	6.04	1.31	
*p*	0.02	0.21	0.30	0.93	
(B)					
<10 ppm	25 (13.6)	3 (10.4)	3 (4.7)	3 (5.4)	34
>10 ppm	13 (24.4)	26 (18.6)	10 (8.3)	12 (9.6)	61
*χ*^2^	14.9	8.17	0.44	1.63	
*p*	0.00	0.00	0.51	0.20	

O: Observed; E: Expected. *p*: probability of (*χ*^2^) of (A) 5 degrees of freedom and (B) 1 degree of freedom. All Some comparisons included a cell size less than five. A Yates’ correction for continuity was applied.
