# Supplementary material for: Characterization of celiac disease related oat proteins: bases for the development of high quality oat varieties suitable for celiac patients
Source: Sci Rep. 2017 Feb 17;7:42588. doi: 10.1038/srep42588 (PMC5314403; doi:10.1038/srep42588)
Supplement: Supplementary Information [file srep42588-s1.pdf]

Characterization of celiac disease related oat proteins: bases for the development of high quality oat varieties suitable for celiac patients

María J. Gimenez[1,*], Ana Real[2], M. Dolores García[1], Carolina Sousa[3], and FranciscoBarro[1]

1 Instituto de Agricultura Sostenible, CSIC, Córdoba, Spain
2 INSERM U1218 ”ACTION”, Bordeaux University, France
3 Departamento de Microbiología y Parasitología, Facultad de Farmacia, Universidad de Sevilla, Sevilla, Spain
* mjga06@ias.csic.es

Supplementary Table S1. Raw data.

ID	Starch (%)	Protein (%)	Monomeric (ug/100mg)	Aggregate (ug/100mg)	Prolamin (ug/100mg)	Non prolamin (ug/100mg)	Gluten (ppm)	Panicle emergence (days)	Height (cm)
AV1	37.07	15.81	22.12	10.97	33.09	127.30	10-20	117	1
AV2	35.19	12.29	16.23	7.10	23.34	101.03	10-20	na	na
AV3	21.18	12.28	9.59	3.11	12.70	110.90	10-20	na	na
AV4	31.44	16.02	18.60	9.93	28.53	133.23	3-10	na	na
AV5	26.92	12.12	13.82	4.49	18.31	103.99	10-20	na	na
AV6	22.06	12.87	8.61	8.02	16.62	113.11	3-10	na	na
AV7	24.05	12.51	12.20	6.16	18.37	107.90	10-20	na	na
AV8	39.38	11.35	16.03	6.48	22.51	92.27	na	na	na
AV9	17.87	10.91	12.92	5.81	18.73	91.53	3-10	na	na
AV10	22.95	13.82	11.36	4.71	16.07	123.03	3-10	na	na
AV11	37.62	13.30	18.01	12.70	30.71	104.81	na	112	1.1
AV12	34.2	14.70	21.07	10.48	31.56	118.05	na	108	1.3
AV13	39.05	7.78	18.63	8.45	27.07	52.96	10-20	106	1.3
AV14	39.27	14.41	24.23	11.15	35.38	111.59	3-10	106	1.5
AV15	39.49	14.54	19.59	8.24	27.84	120.20	3-10	102	1.3
AV16	40.04	15.84	21.89	8.72	30.61	130.43	na	107	1.2
AV17	42.69	16.42	24.55	13.25	37.80	129.72	30-40	109	1.3
AV18	39.38	16.83	24.69	14.93	39.62	132.10	na	111	1.4
AV19	39.16	15.46	20.60	11.19	31.78	125.53	na	113	1.3
AV20	30.01	18.62	20.64	11.04	31.68	157.19	10-20	118	1.4
AV21	35.96	17.96	21.56	12.56	34.13	148.21	10-20	113	1.4
AV22	27.47	20.02	22.30	12.26	34.56	168.34	10-20	118	1.5
AV23	34.31	19.25	24.78	8.60	33.38	161.75	3-10	116	1.5
AV24	42.36	16.10	18.35	9.18	27.53	135.46	3-10	100	1.1
AV25	43.35	14.49	22.70	13.34	36.04	111.41	na	100	1.25
AV26	33.6	18.05	24.95	11.80	36.75	146.35	10-20	118	1.3
AV27	41.26	15.23	22.60	13.59	36.19	118.49	na	108	1.2
AV28	44.79	16.72	23.66	11.91	35.58	134.01	10-20	118	1.2
AV29	43.79	14.62	21.69	13.27	34.96	113.62	10-20	113	1.6
AV30	39.16	17.18	20.30	13.31	33.62	140.47	20-30	124	1.6
AV31	39.27	12.06	12.89	10.76	23.64	98.57	30-40	126	1.6
AV32	38.39	15.79	12.26	10.14	22.40	136.99	10-20	109	1.4
AV33	43.02	18.17	17.66	12.91	30.57	153.73	10-20	117	1.55
AV34	40.93	14.22	13.59	6.77	20.36	123.57	na	107	1.2
AV35	45.89	16.33	21.93	12.31	34.23	132.40	10-20	126	1.5
AV36	40.93	17.15	20.04	7.73	27.78	146.67	10-20	109	1.35
AV37	39.49	16.99	18.86	12.18	31.04	142.06	20-30	122	1.7
AV38	42.36	16.32	19.24	11.36	30.60	135.66	na	124	1.6
AV39	34.53	18.17	23.30	6.11	29.42	155.26	10-20	122	1.7
AV40	40.82	17.29	22.35	12.09	34.44	141.85	10-20	107	1.35
AV41	42.8	17.02	20.77	16.21	36.98	136.87	10-20	117	1.4
AV49	44.46	15.72	20.70	15.22	35.92	124.56	3-10	107	1.25
AV50	43.79	15.61	19.17	14.34	33.50	125.77	10-20	107	1.25
AV51	44.68	17.53	23.30	15.90	39.20	139.67	na	112	1.2
AV52	43.13	12.79	25.06	13.41	38.47	93.02	3-10	112	1.2
AV53	41.04	14.48	25.78	13.94	39.72	108.49	na	112	1.1
AV54	40.38	16.38	18.83	14.80	33.63	133.07	3-10	109	1.1
AV55	43.91	16.85	25.14	16.73	41.87	130.17	<3	109	1.15
AV56	44.68	17.75	25.45	13.08	38.53	142.22	30-40	112	1.4
AV57	46.44	16.18	26.76	12.62	39.38	125.76	30-40	107	1.2
AV58	44.79	14.10	20.60	11.68	32.28	111.42	3-10	107	1.3
AV59	40.71	16.42	15.81	15.85	31.66	135.12	3-10	111	1.4
AV60	34.2	18.51	25.19	11.79	36.98	151.20	10-20	114	1.5
AV61	34.53	18.43	22.59	10.32	32.91	154.11	10-20	114	1.5
AV62	38.39	16.74	22.80	11.46	34.27	135.97	<3	112	1.5
AV63	40.04	19.64	28.95	12.02	40.97	158.88	na	114	1.5
AV64	34.42	18.90	23.77	12.22	35.98	155.94	na	116	1.5
AV65	42.58	15.13	21.97	11.79	33.76	120.28	3-10	109	1.4
AV66	35.08	17.16	23.07	10.90	33.98	140.61	na	109	1.3
AV67	44.46	14.58	20.98	10.62	31.60	117.25	10-20	107	1.35
AV68	26.81	16.32	19.99	8.67	28.66	137.33	20-30	124	1.6
AV69	35.08	16.89	19.62	11.04	30.66	141.30	na	116	1.4
AV70	28.35	18.59	20.33	7.30	27.63	160.86	10-20	116	1.4
AV71	32.98	15.95	19.24	6.95	26.19	135.87	10-20	116	1.5
AV72	37.29	14.55	19.61	9.69	29.30	118.89	na	107	1.3
AV73	28.68	18.10	22.54	7.57	30.10	153.92	20-30	122	1.6
AV74	27.25	19.35	21.16	6.57	27.73	168.62	3-10	122	1.6
AV75	26.7	19.42	24.51	9.27	33.78	163.87	na	113	1.45
AV76	25.7	15.38	19.38	6.25	25.62	130.72	10-20	122	1.6
AV77	31.66	17.89	22.44	9.22	31.66	150.28	na	116	1.6

Supplementary Table S1. Raw data.

ID	Starch (%)	Protein (%)	Monomeric (ug/100mg)	Aggregate (ug/100mg)	Prolamin (ug/100mg)	Non prolamin (ug/100mg)	Gluten (ppm)	Panicle emergence (days)	Height (cm)
AV78	37.18	19.29	23.35	10.32	33.67	162.42	<3	109	1.5
AV79	30.01	19.03	23.11	7.87	30.98	162.27	na	116	1.45
AV80	37.29	16.08	26.60	11.63	38.23	126.08	na	101	1.15
AV81	39.05	16.17	22.09	12.01	34.10	130.86	na	109	1.4
AV82	36.96	16.33	18.84	8.59	27.43	138.54	>40	112	1.3
AV83	38.94	14.50	13.76	7.43	21.19	125.95	3-10	112	1.6
AV84	40.26	16.82	24.13	11.06	35.20	136.70	10-20	114	1.2
AV85	41.26	14.93	20.46	8.05	28.51	123.79	3-10	114	1.4
AV86	39.38	17.39	24.13	10.34	34.47	143.02	20-30	114	1.4
AV87	37.18	14.91	20.01	7.79	27.80	124.19	3-10	107	1.4
AV88	40.71	16.50	20.23	8.25	28.48	139.43	na	107	1.45
AV89	40.93	17.76	22.12	10.12	32.25	148.61	20-30	114	1.5
AV90	39.93	16.12	24.66	9.02	33.69	130.89	10-20	112	1.3
AV91	44.9	16.24	25.48	9.66	35.13	130.83	na	108	1.5
AV92	43.13	13.95	19.11	7.71	26.82	115.35	30-40	109	1.4
AV93	36.4	17.44	25.71	10.74	36.45	141.62	10-20	112	1.4
AV94	38.94	15.85	23.23	12.25	35.49	126.35	na	107	1.3
AV95	44.68	15.78	20.21	10.81	31.02	129.88	3-10	109	1.23
AV96	44.57	15.37	21.12	9.76	30.88	125.81	3-10	100	0.95
AV97	41.35	14.55	19.29	9.72	29.01	119.21	30-40	109	1.3
AV98	35.3	15.70	20.66	11.94	32.60	127.73	3-10	109	1.4
AV99	39.27	14.71	23.95	12.50	36.44	114.31	3-10	101	1.2
AV100	41.87	17.19	23.25	12.01	35.26	140.14	na	112	1.5
AV101	40.82	18.43	15.04	14.13	29.18	158.16	na	109	1.5
AV102	32.76	17.06	18.25	10.81	29.06	144.50	3-10	122	1.6
AV103	36.62	16.67	20.88	12.66	33.54	136.54	20-30	114	1.5
AV104	41.92	16.07	22.03	13.29	35.32	128.87	3-10	107	1.35
AV105	36.29	19.62	17.44	17.78	35.22	164.57	10-20	113	1.4
AV106	40.6	16.22	23.16	13.20	36.36	129.46	na	93	1.1
AV107	40.93	15.91	21.75	8.13	29.88	136.31	3-10	108	1.3
AV108	40.6	19.31	23.20	9.39	32.58	163.70	20-30	114	1.4
AV109	36.41	16.79	23.21	11.04	34.25	137.06	20-30	122	1.1
AV110	28.9	14.89	17.65	9.86	27.50	123.46	na	107	1.35
AV111	39.6	15.84	16.25	10.72	26.96	133.61	20-30	114	1.5
AV112	38.28	15.17	18.88	12.26	31.13	122.91	30-40	116	1.5
AV113	42.02	13.98	18.60	8.57	27.17	114.70	3-10	107	1.4
AV114	38.72	15.22	23.07	11.62	34.69	120.18	na	107	1.3
AV115	29.23	18.29	21.12	9.40	30.52	154.64	10-20	118	1.1
AV116	37.4	16.94	22.44	10.61	33.05	139.09	10-20	114	1.4
AV117	39.93	17.39	21.22	7.46	28.69	147.48	20-30	114	1.5
AV118	42.25	15.95	23.27	8.65	31.93	130.01	20-30	112	1.6
AV119	38.5	15.40	21.83	8.54	30.37	125.85	3-10	110	1.4
AV120	40.6	18.19	26.08	12.26	38.34	146.61	na	112	1.7
AV121	33.76	18.37	19.66	10.38	30.04	156.27	10-20	112	1.6
AV122	35.19	17.54	21.74	13.64	35.38	143.10	10-20	112	1.3
AV123	33.54	15.85	10.30	9.12	19.41	140.73	3-10	109	1.4
AV124	35.08	17.46	24.97	13.03	38.00	139.83	10-20	113	1.3
AV125	33.31	17.85	23.36	10.70	34.06	147.28	10-20	116	1.5
AV126	38.83	17.55	18.85	13.81	32.66	145.61	10-20	107	1.5
AV127	31.11	19.19	24.28	8.60	32.88	161.90	20-30	114	1.5
AV128	35.52	17.71	21.72	11.90	33.62	146.40	na	116	1.5
AV129	32.43	16.82	21.50	11.12	32.62	138.21	10-20	106	1.5
AV130	40.6	16.21	23.11	11.87	34.98	129.86	na	107	1.25
AV131	39.93	15.62	19.12	10.37	29.49	129.15	na	107	1.25
AV132	38.5	15.77	22.04	10.36	32.40	127.90	10-20	108	1.4
AV133	36.18	14.03	19.13	13.07	32.20	110.73	na	104	1.7
AV134	36.73	14.11	21.39	12.53	33.91	109.91	<3	107	1.25
AV135	33.98	13.09	17.43	11.86	29.28	103.95	na	92	1.2
AV136	36.4	15.50	21.37	10.23	31.60	125.93	3-10	109	1.4
AV137	36.4	14.65	18.39	11.71	30.10	118.80	<3	101	1.3
AV138	40.38	13.83	19.95	11.36	31.31	109.44	<3	107	1.2
AV139	26.14	11.26	11.44	3.74	15.18	98.39	30-40	na	na

Supplentary Table S2. Accesion codes and information of the plant material used in this study.

ID	acce_numb/cv_name	Country of Origin*	Year	Type
AV1	Frigante	FR	ND	Commercial cultivar
AV2	Hamel	ES, FR, IT	ND	Commercial cultivar
AV3	Canyon	DK, DE, UK	ND	Commercial cultivar
AV4	Alcudia	ES	ND	Commercial cultivar
AV5	Chimene	ES, FR	ND	Commercial cultivar
AV6	Efesos	AT	ND	Commercial cultivar
AV7	Scorpion	CZ, DK, DE, EE, LT, LVDK	ND	Commercial cultivar
AV8	Rapidena	ES	ND	Commercial cultivar
AV9	Primula	IT	ND	Commercial cultivar
AV10	Chappline	FR	ND	Commercial cultivar
AV11	PI 258563	Tunisia	1959	Landrace
AV12	PI 258566	Greece	1959	Landrace
AV13	PI 258569	Italy	1959	Landrace
AV14	PI 258570	Italy	1959	Landrace
AV15	PI 258571	Spain	1959	Landrace
AV16	PI 258572	Spain	1959	Landrace
AV17	PI 258579	Greece	1959	Landrace
AV18	PI 258580	Greece	1959	Landrace
AV19	PI 258584	Greece	1959	Landrace
AV20	Clav 1013	USA	1920	Landrace
AV21	Clav 1012	USA	1920	Developed cultivar
AV22	Clav 1011	USA	1920	Landrace
AV23	Clav 1010	USA	1920	Landrace
AV24	Clav 1009	USA	1920	Developed cultivar
AV25	Clav 1008	USA	1920	Landrace
AV26	Clav 1007	USA	1920	Landrace
AV27	Clav 1006	USA	1920	Landrace
AV28	Clav 362	Spain	1904	Landrace
AV29	Clav 357	Greece	1904	Landrace
AV30	PI 51385	Spain	1920	Landrace
AV31	PI 51384	Spain	1920	Landrace
AV32	PI 51383	Spain	1920	Landrace
AV33	PI 51382	Spain	1920	Landrace
AV34	PI 51305	Spain	1920	Landrace
AV35	PI 51304	Spain	1920	Landrace
AV36	PI 51303	Spain	1920	Landrace
AV37	PI 51302	Spain	1920	Landrace
AV38	PI 51301	Spain	1920	Landrace
AV39	PI 51300	Spain	1920	Landrace
AV40	PI 158209	Spain	1947	Landrace
AV41	PI 157883	Italy	1947	Landrace
AV49	PI 110259	Italy	1935	Landrace
AV50	PI 264427	Greece	1960	Developed cultivar
AV51	PI 264838	Greece	1960	Landrace
AV52	PI 264839	Greece	1960	Landrace
AV53	PI 264840	Greece	1960	Landrace
AV54	PI 264841	Greece	1960	Landrace
AV55	PI 287264	Greece	1963	Landrace
AV56	PI 287265	Greece	1963	Landrace
AV57	PI 287266	Greece	1963	Landrace
AV58	PI 287267	Greece	1963	Landrace
AV59	PI 287269	Greece	1963	Landrace
AV60	PI 110258	Italy	1935	Landrace
AV61	PI 110257	Italy	1935	Landrace
AV62	PI 110255	Italy	1935	Landrace
AV63	PI 110254	Italy	1935	Landrace
AV64	PI 61999	Italy	1924	Landrace
AV65	PI 55521	Tunisia	1922	Landrace
AV66	PI 55523	Tunisia	1922	Landrace
AV67	PI 55524	Tunisia	1922	Landrace
AV68	PI 58042	Spain	1923	Landrace
AV69	PI 51386	Spain	1920	Landrace
AV70	Clav 1030	USA	1920	Landrace

Supplentary Table S2. Accesion codes and information of the plant material used in this study.

ID	acce_numb/cv_name	Country of Origin*	Year	Type
AV71	Clav 1027	USA	1920	Landrace
AV72	Clav 1026	USA	1920	Landrace
AV73	Clav 1025	USA	1920	Landrace
AV74	Clav 1024	USA	1920	Developed cultivar
AV75	Clav 1021	USA	1920	Landrace
AV76	Clav 1018	USA	1920	Landrace
AV77	Clav 1016	USA	1920	Landrace
AV78	Clav 1015	USA	1920	Developed cultivar
AV79	Clav 1014	USA	1920	Landrace
AV80	PI 258585	Greece	1959	Landrace
AV81	PI 219764	Greece	1954	Landrace
AV82	PI 219763	Greece	1954	Landrace
AV83	PI 219762	Greece	1954	Landrace
AV84	PI 219761	Greece	1954	Landrace
AV85	PI 198908	Italy	1951	Landrace
AV86	PI 219760	Greece	1954	Landrace
AV87	PI 189767	Tunisia	1950	Landrace
AV88	PI 189766	Tunisia	1950	Landrace
AV89	PI 158229	Spain	1947	Landrace
AV90	PI 219765	Greece	1954	Landrace
AV91	PI 221276	Italy	1954	Landrace
AV92	PI 221277	Italy	1954	Landrace
AV93	PI 249721	Greece	1958	Landrace
AV94	PI 249932	Greece	1958	Landrace
AV95	PI 258548	Italy	1959	Landrace
AV96	PI 253347	Spain	1958	Landrace
AV97	PI 258560	Tunisia	1959	Landrace
AV98	PI 258561	Tunisia	1959	Landrace
AV99	PI 249933	Greece	1958	Landrace
AV100	Clav 1050	USA	1920	Landrace
AV110	PI 51199	Spain	1920	Landrace
AV120	Clav 2866	Italy	1930	Landrace
AV130	PI 287293	Italy	1963	Landrace
AV101	Clav 1049	USA	1920	Landrace
AV102	Clav 1046	USA	1920	Landrace
AV103	Clav 1041	USA	1920	Landrace
AV104	Clav 1039	USA	1920	Developed cultivar
AV105	Clav 1038	USA	1920	Landrace
AV106	Clav 1035	USA	1920	Developed cultivar
AV107	Clav 1034	USA	1920	Landrace
AV108	Clav 1033	USA	1920	Landrace
AV109	Clav 1031	USA	1920	Landrace
AV111	PI 51299	Spain	1920	Landrace
AV112	PI 51298	Spain	1920	Landrace
AV113	PI 51203	Spain	1920	Landrace
AV114	PI 51202	Spain	1920	Landrace
AV115	PI 25854	Spain	1909	Landrace
AV116	PI 25851	Spain	1909	Landrace
AV117	PI 25850	Spain	1909	Landrace
AV118	Clav 9109	Spain	1971	Landrace
AV119	Clav 9100	Greece	1971	Landrace
AV121	Clav 2864	Italy	1930	Developed cultivar
AV122	Clav 1822	Spain	1920	Landrace
AV123	Clav 1820	Spain	1920	Landrace
AV124	Clav 1060	USA	1920	Landrace
AV125	Clav 1058	USA	1920	Developed cultivar
AV126	Clav 1057	USA	1920	Landrace
AV127	Clav 1054	USA	1920	Developed cultivar
AV128	Clav 1052	USA	1920	Landrace
AV129	Clav 1051	USA	1920	Landrace
AV131	PI 338516	Greece	1968	Landrace
AV132	PI 338517	Greece	1968	Landrace
AV133	PI 411398	Tunisia	1975	Landrace

Supplentary Table S2. Accesion codes and information of the plant material used in this study.

ID	acce_numb/cv_name	Country of Origin*	Year	Type
AV134	PI 564724	Italy	1993	Developed cultivar
AV135	PI 564725	Italy	1993	Developed cultivar
AV136	PI 564726	Italy	1993	Developed cultivar
AV137	PI 158213	Tunisia	1947	Landrace
AV138	Prevision	ES	ND	Commercial cultivar
AV139	Aintree	ES	ND	Commercial cultivar

*The origin of the commercial cultivars is that indicated in the *Common catalogue of varieties of agricultural plant species* (http://ec.europa.eu/food/plant/propagation/catalogues/agri2011/73.html)

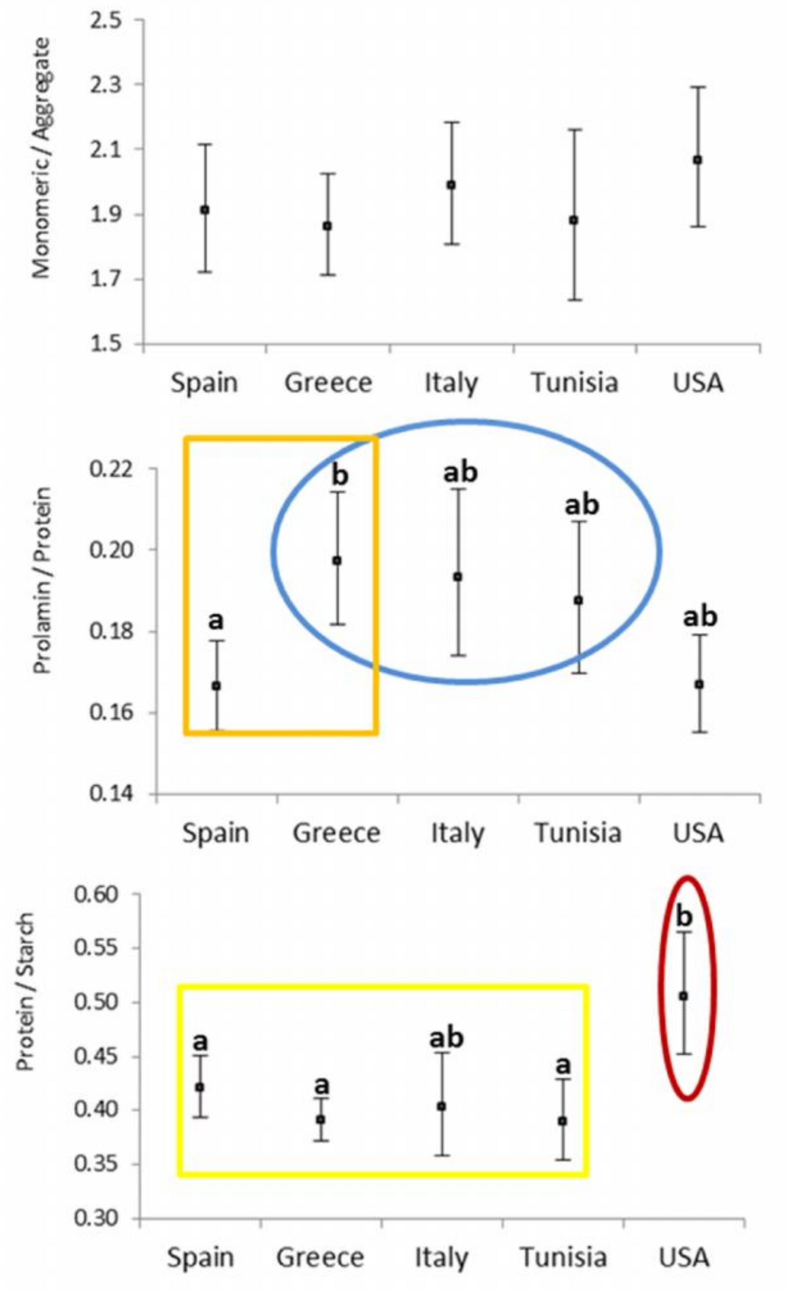

Supplementary Figure S1. Means and 95% CIs for flour quality composition ratios of oat accessions grouped by their origin. Yellow rectangles indicate no significant differences among Mediterranean accessions; Blue ovals and orange squares highlight differences between Spanish and Greek accessions. Red ovals indicate that a mean is significantly different from at least other three group means

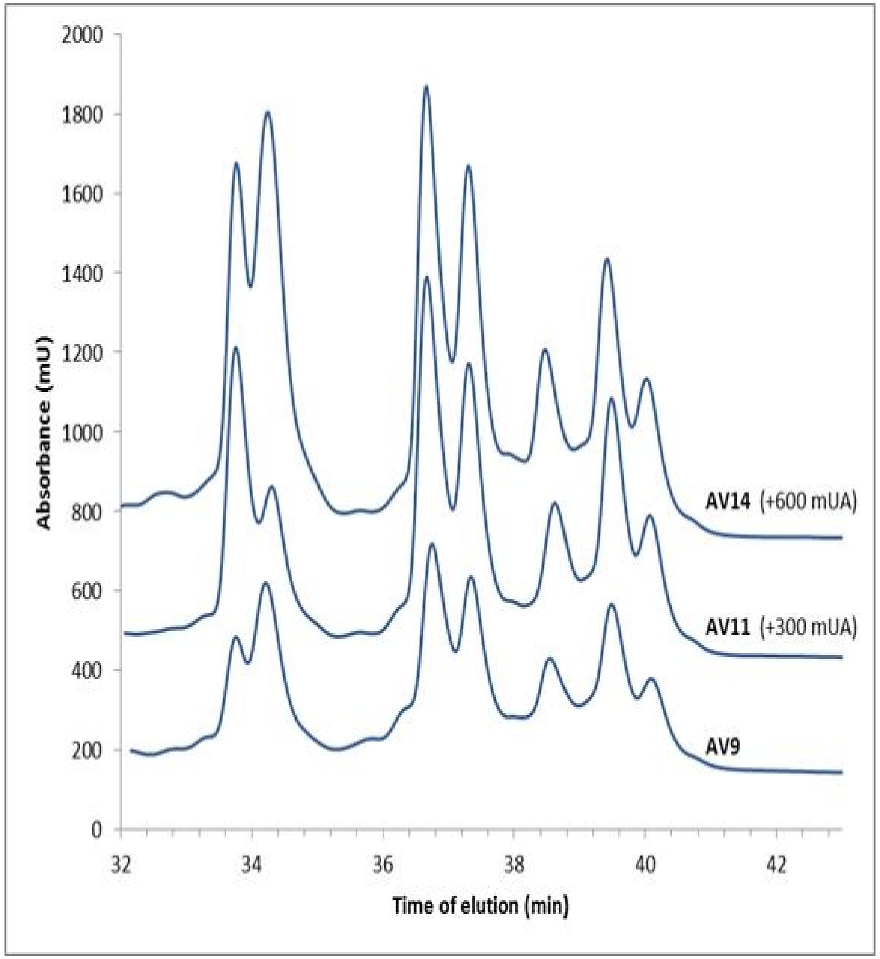

Supplementary Figure S2. Chromatograms of three oat accessions with the same pattern of peaks differing in relative peak height

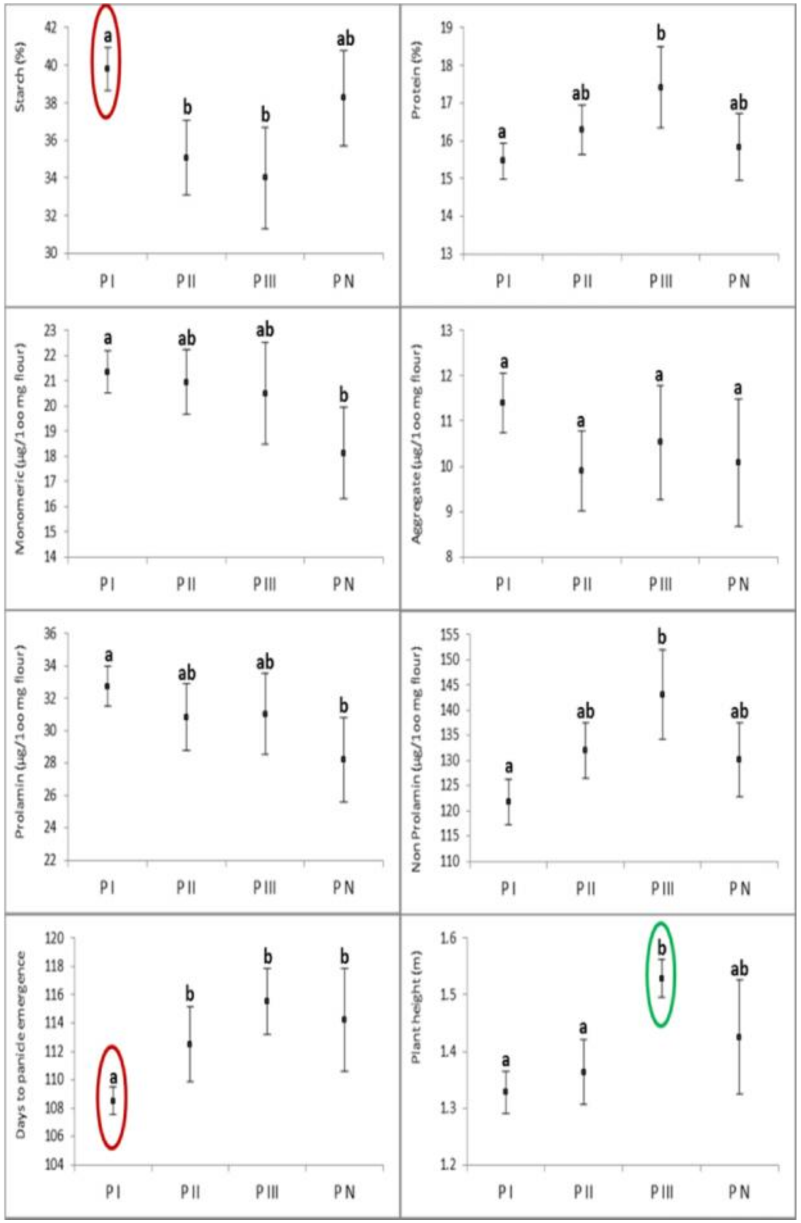

Supplementary Figure S3. Means and 95% CIs for plant features and flour composition of oat accessions grouped by their RP-HPLC pattern. Colored ovals indicate a significant difference between the mean of one group of lines and at least two other groups.

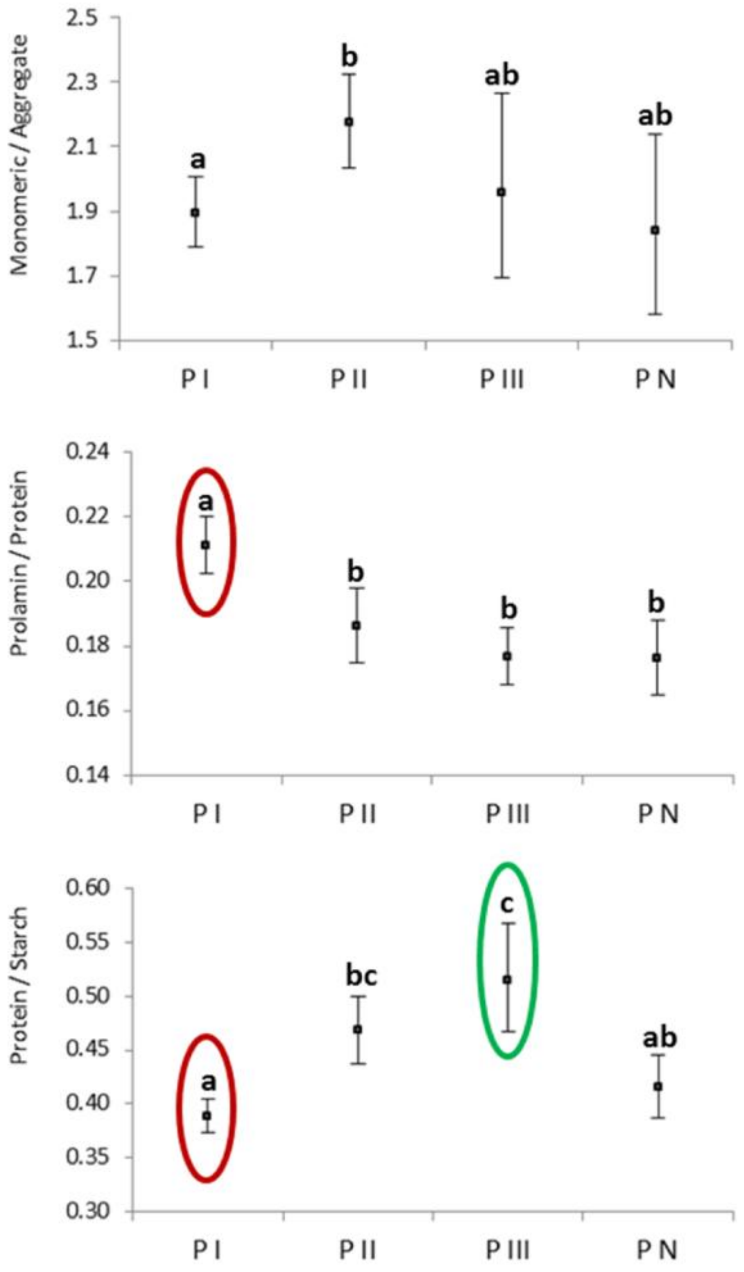

Supplementary Figure S4. Means and 95% CIs for flour quality composition ratios of oat accessions grouped by their RP-HPLC patterns. Colored ovals indicate a significant difference between the mean of one group of lines and at least two other groups.
